# Regional Variations in Melanoma Histologic Characteristics: A Multicenter Cohort Study from Two Southeastern European Countries

**DOI:** 10.3390/jcm15166461

**Published:** 2026-08-20

**Authors:** Jelena Jeremić, Branko Suđecki, Miloš Bojić, Zoran Bukumirić, Zorka Inić, Milan Jovanović, Milan Stojičić, Zoran Terzić, Marinko Paunović, Mileta Golubović, Marko S. Jović

**Affiliations:** 1 Clinic for Burns, Plastic and Reconstructive Surgery, University Clinical Center of Serbia, 11000 Belgrade, Serbia; 2 Department of Surgery, Faculty of Medicine, University of Belgrade, 11000 Belgrade, Serbia; 3 Clinic for Burns, Plastic and Reconstructive Surgery, Clinical Center of Montenegro, 81000 Podgorica, Montenegro; 4 Faculty of Medicine, University of Montenegro, 81000 Podgorica, Montenegro; 5 Institute for Medical Statistics, School of Medicine, University of Belgrade, 11000 Belgrade, Serbia; 6 Department of Clinical Research, Faculty of Health Sciences, University of Southern Denmark, 5230 Odense, Denmark; 7 Institute for Oncology and Radiology of Serbia, 11000 Belgrade, Serbia; 8 Center for Pathology, Clinical Center of Montenegro, 81000 Podgorica, Montenegro

**Keywords:** cutaneous melanoma, diagnosis, Breslow thickness, multicenter study, healthcare accessibility

## Abstract

**Background/Objectives**: Cutaneous melanoma prognosis is largely determined at diagnosis, with Breslow thickness, subtype and other histopathological aspects being key prognostic indicators. Variations in healthcare organization and environmental factors may influence melanoma presentation. This study aimed to compare epidemiological, clinical, and histopathological characteristics of primary cutaneous melanoma between two national referral centers in Southeastern Europe. **Methods**: A multicenter retrospective cohort study was conducted at the University Clinical Center of Serbia (Belgrade) and the Clinical Center of Montenegro (Podgorica). Consecutive patients treated for primary cutaneous melanoma between January 2018 and December 2022 were included. Data of interest were extracted from medical records. Annual regional sunshine hours were obtained from publicly available climatological databases. Group comparisons were performed using appropriate parametric and non-parametric tests, followed by a multivariable ordinal logistic regression analysis. **Results**: A total of 412 patients were analyzed. Age and sex distributions were comparable between cohorts. Nodular melanoma predominated in Montenegro, whereas superficial spreading melanoma was most common in Belgrade (*p* < 0.001). Median Breslow thickness was significantly higher in Montenegro (*p* = 0.001), with a greater proportion of tumors >4 mm (*p* = 0.001). In multivariable analysis, increasing age (*p* < 0.001), male sex (*p* = 0.027), and treatment in Montenegro (*p* = 0.001) were independently associated with higher Breslow thickness categories. **Conclusions**: Patients treated in Montenegro presented with thicker melanomas and a higher proportion of nodular subtype. These differences may reflect multifactorial influences, including healthcare accessibility and diagnostic pathways. Further prospective studies incorporating other histopathological aspects, time-to-diagnosis and survival outcomes are warranted.

## 1. Introduction

### 1.1. Epidemiology of Melanoma

Cutaneous melanoma is the most malignant skin cancer, with a disproportionate share of skin cancer-related mortality worldwide [1]. According to GLOBOCAN, 57,043 melanoma-related deaths were recorded globally in 2020 [2]. In Europe, melanoma accounts for approximately 1.4% of all cancer-related deaths, claiming nearly 20,000 lives annually [3,4]. Melanoma burden varies considerably across populations. Epidemiological data indicate that higher rates are primarily reported in Australia, North America, and Europe, with greater prevalence observed among older age groups and male patients [5,6,7,8]. Southeastern Europe is characterized by low reported incidence but disproportionately high mortality-to-incidence ratio, when compared to Western and Northern Europe [9]. In Serbia, 723 new melanoma cases were registered in 2022 (age-standardized incidence rate 6.0/100,000, world standard population), while melanoma-related deaths totaled 256 in the same year (standardized mortality rate 1.8/100,000) [10]. Notably, Montenegro, together with Croatia, Slovenia, and Norway, has been reported to have mortality rates nearly double the European average [10,11].

### 1.2. Ultraviolet Radiation Exposure

Extended exposure to ultraviolet radiation (UVR), whether from natural sunlight or artificial sources such as indoor tanning devices, is considered a major etiological factor in melanoma development [7]. Additional established risk factors include a family history of invasive cutaneous melanoma among first-degree relatives, prior diagnosis of primary invasive melanoma, a high burden of benign melanocytic nevi, the presence of multiple clinically atypical (dysplastic) nevi, lighter skin phototypes (I–II), light-colored hair, a history of severe sunburns, use of tanning beds at younger ages, and occupational or environmental exposure to pesticides [8]. Nevus count shows a strong, dose-dependent association, while the presence of atypical (dysplastic) naevi confers a similarly strong risk [12]. Family history confers a moderate-to-strong risk, while evidence regarding cumulative vs. intermittent ultraviolet exposure remains comparatively heterogeneous across studies [13]. In response, various population-based prevention strategies have been implemented. Primary prevention efforts focus on reducing UVR exposure through public education campaigns, the use of broad-spectrum sunscreen and protective clothing, behavioral modification, and regulation of tanning devices and sunscreen labeling [14]. Secondary prevention strategies emphasize early detection, including total-body skin examinations and awareness initiatives promoting self-examination [15,16,17]. However, disparities in melanoma incidence and stage at diagnosis persist across Europe, reflecting differences in public awareness, healthcare accessibility, and the organization of primary and secondary prevention programs.

### 1.3. Melanoma Prevention

Early detection remains pivotal for improving melanoma-specific survival [18,19]. Timely recognition of suspicious lesions, patient education on skin self-examination, and prompt referral to dermatologic specialists are essential components of effective melanoma control [20,21,22]. The Euromelanoma campaign represents one of Europe’s most prominent melanoma prevention and screening initiatives, promoting awareness and early diagnosis through coordinated nationwide activities. Serbia has participated in the Euromelanoma campaign since 2008, and Montenegro joined in 2013, reflecting regional engagement in structured awareness efforts towards this public health issue [23]. Although both Serbia and Montenegro participate in the Euromelanoma campaign, population screening initiatives of this kind have shown variable detection yields across participating European countries, with melanoma suspicion rates in Euromelanoma cohorts ranging widely depending on population risk profile and screening uptake [24].

### 1.4. Prognosis

Prognosis of primary cutaneous melanoma is strongly determined at the time of diagnosis, with tumor thickness, measured by Breslow depth, remaining the most reproducible histopathological predictor of survival. Furthermore, histopathological subtype strongly influences melanoma behavior and clinical presentation. Superficial spreading melanoma (SSM) typically shows a prolonged radial growth phase that enables earlier detection. In contrast, nodular melanoma (NM) is characterized by rapid vertical growth and early invasion, and is more often diagnosed at greater Breslow thickness, contributing to poorer outcomes [25]. Microsatellitosis has also been associated with an increased risk of nodal metastasis and is incorporated into current AJCC staging for stage III disease, independent of clinically detected nodal involvement [26].

Although regional incidence and mortality trends have been described, detailed comparative analyses of histopathological characteristics across neighboring healthcare systems are limited. Evaluating differences between centralized national systems and large metropolitan tertiary centers may provide insight into system-level determinants of melanoma presentation. This study aimed to compare epidemiological, clinical and histopathological characteristics of primary cutaneous melanoma between two national referral melanoma centers. To date, no study has directly compared histopathological melanoma characteristics between these two neighboring countries.

## 2. Materials and Methods

### 2.1. Data Design, Exclusion Criteria and Extraction Process

A multicentric retrospective cohort study was conducted at the Clinic for Burns, Plastic and Reconstructive Surgery, University Clinical Center of Serbia in Belgrade, Serbia, and the Clinic for Plastic and Reconstructive Surgery, Clinical Center of Montenegro in Podgorica, Montenegro. Both institutions are referral melanoma centers in their respective countries with sufficient patient volumes for this type of study. The study sample consisted of consecutive patients treated for primary cutaneous melanoma at either center in the period between January 2018 and December 2022. All patients at both institutions were treated according to the current American Joint Committee on Cancer (AJCC, 8th edition) and National Comprehensive Cancer Network (NCCN) guidelines and underwent standardized surgical management beginning with excisional biopsy of the primary lesion. Definitive wide local excision margins were determined according to Breslow thickness, consistent with AJCC/NCCN recommendations (0.5–1 cm for in situ disease, 1 cm for melanomas ≤1 mm, 1–2 cm for melanomas 1.01–2 mm, and 2 cm for melanomas >2 mm in thickness). Subsequent management, including sentinel lymph node biopsy, complete lymph node dissection, or referral for adjuvant systemic therapy, was determined according to tumor stage and national guideline recommendations at each center. Histopathological samples at both institutions were analyzed by a pathologist experienced in melanocytic lesions. Formal double-reading or interobserver variability testing was not performed. Patients were excluded if they had recurrent melanoma, metastatic melanoma without a clearly identifiable primary lesion, mucosal or ocular melanoma, or if any required data of interest were missing. Extracted variables included basic demographic data (age, sex, place of residence, treatment institution), clinical localization of primary lesion, and histopathologic features (melanoma subtype and Breslow thickness). Data were obtained from medical records. Breslow thickness was categorized according to AJCC (≤1 mm, 1.01–2.0 mm, 2.01–4.0 mm, and >4.0 mm). Annual average sunshine hours for each region were obtained from publicly available climatological data provided by Climate-Top, an online database compiling long-term meteorological averages for cities and regions worldwide [27]. All UCCS patients were assigned the Belgrade sunshine-hour value, and all CCM patients were assigned the Podgorica value, reflecting the predominant catchment area.

### 2.2. Statistical Analysis

Statistical analyses were presented as frequency (percent), median (range), or mean ± standard deviation, as appropriate. Differences between groups for continuous variables were tested using the independent samples *t*-test or Mann–Whitney U test, as appropriate. Differences in categorical variables were assessed by chi-square test or Fisher’s exact test, as appropriate. The impact of variables of interest on Breslow thickness categories was evaluated using multivariable ordinal logistic regression. The multivariable model included age, sex, institution, and anatomical localization as covariates. A two-tailed *p*-value <0.05 was considered statistically significant. All analyses were conducted using IBM SPSS Statistics version 24 (IBM Corporation, Armonk, NY, USA).

## 3. Results

### 3.1. Epidemiology

The study included a total of 412 patients, of whom 55.6% were treated at the University Clinical Center of Serbia (UCCS) and 44.4% at the Clinical Center of Montenegro (CCM). The mean age was 63.1 ± 15.5 years in the UCCS and 62.7 ± 17.3 years in the CCM cohort. In both groups, the majority of patients were in the 61–80-year age category (52.4% and 46.4%, respectively), with no significant difference in overall age distribution between the regions (*p* = 0.228). Male patients were slightly predominant in both cohorts, and no significant differences were observed in age or sex distribution between the two groups (*p* = 0.80 and *p* = 0.92, respectively) (Table 1 and Table 2). Based on available climatological data, Podgorica had significantly more annual sunny hours compared with Belgrade (median 2238 vs. 2112 h; *p* < 0.001).

### 3.2. Clinical and Histopathological Data

The distribution of clinical tumor localization did not differ significantly between the cohorts (*p* = 0.970), with the trunk being the most common site in both groups (38.4% vs. 37.2%, respectively) (Table 2). A statistically significant difference was observed in the distribution of histological melanoma subtypes between the two cohorts (*p* < 0.001). Superficial spreading melanoma was the most frequent subtype in the Belgrade cohort (65.6%), whereas nodular melanoma predominated in the Montenegrin cohort (54.7%). The Montenegrin cohort showed a significantly greater median Breslow thickness compared with the UCCS cohort (3 mm vs. 2 mm; *p* = 0.001). In addition, the overall distribution of Breslow thickness categories differed significantly between the groups, with a higher proportion of melanomas measuring > 4 mm in thickness in the Montenegrin cohort (37.7% vs. 28.4%; *p* = 0.001) (Table 2).

**Figure 1:**
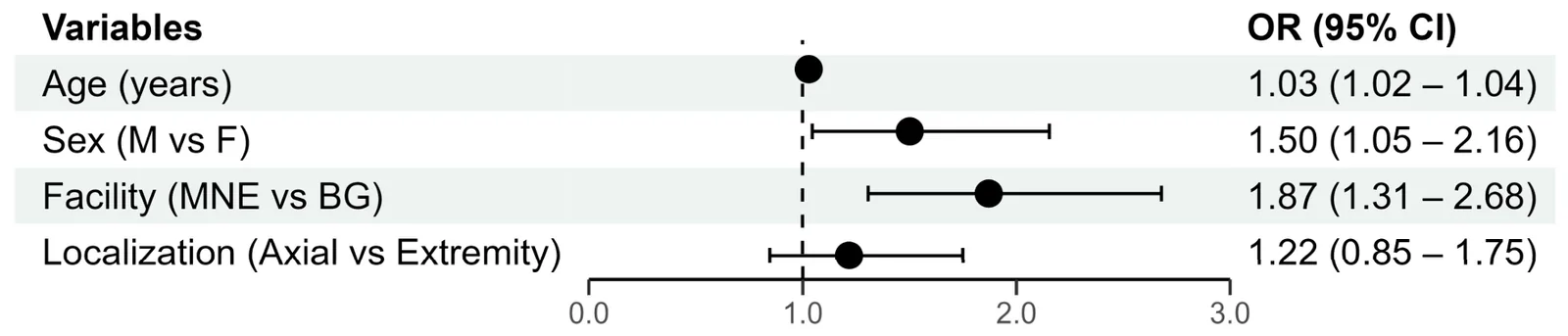
Forest plot illustrates the results of multivariable ordinal logistic regression analysis for predictors of higher Breslow thickness categories; OR—odds ratios, CI—confidence intervals, M—male, F—female, MNE—Montenegro, BG—Belgrade.

Clinically relevant variables and/or those showing a significant association with Breslow thickness in univariate analysis were included in a multivariable ordinal logistic regression model. Increasing age, male sex, and treatment in Montenegro were independently associated with higher Breslow thickness categories in the multivariable analysis (*p* < 0.001, *p* = 0.027, and *p* = 0.001, respectively). Localization was not significantly associated with Breslow thickness category in the multivariable model (*p* = 0.287) (Table 3, Figure 1).

## 4. Discussion

This multicenter retrospective cohort study involved two national referral melanoma centers, with broadly comparable demographic profiles between the two groups, contributing to internal validity of between-center comparisons. In both cohorts, the dominant age group was 61–80 years, aligning with the established patterns of melanoma risk rising with age and a tendency toward higher rates among older males [28,29]. Based on these findings, our cohorts matched well for comparisons of clinical lesion presentation as well as histopathologic profiles such as subtype and Breslow thickness. The study results found that Montenegrin cohort presented with a significantly higher proportion of nodular melanomas and greater Breslow thickness at diagnosis time, when compared to the Belgrade cohort. In the multivariable analysis, older age, male sex, and Montenegro treatment facility remained associated with greater Breslow thickness, whereas anatomical localization was not.

### 4.1. Sunshine Hours per Region

Although Podgorica had significantly higher annual sunshine hours than Belgrade based on the available climatologic data, interpreting this variable as a direct proxy for biologically relevant UV dose requires caution. The melanoma–UVR relationship is complex and strongly influenced by the host’s susceptibility, behavior, and the spectral composition of UV exposure. UVB is substantially more genotoxic per photon than UVA, yet UVA exposure is more abundant environmentally and varies with season, latitude, and altitude [30,31]. Furthermore, several meta-analyses indicate inconsistent associations of “chronic” sun exposure with melanoma, possibly due to changes in effect due to sun sensitivity, phenotype, and heterogeneous exposure measurement [32,33]. Similarly, Chang et al. reported in a pooled analysis that total sun exposure does not necessarily increase melanoma risk at all body sites. Associations vary by latitude and anatomic site, with weak or absent associations for trunk melanomas in particular [34]. It should also be noted that sunshine-hour values were assigned based on each center’s predominant catchment area (Belgrade and Podgorica) rather than each patient’s individual place of residence. Therefore, this finding should be taken with caution in further interpretation of the study’s results. It is also plausible that a substantial proportion of the Montenegrin cohort resides in or spends considerable time in the country’s coastal region, a major domestic and touristic destination associated with high seasonal recreational sun exposure. If true, this pattern of intense intermittent UV exposure could represent a genuine, individual-level contributor to melanoma risk and presentation in this cohort. This remains speculative, as individual-level sun-exposure behavior data were not collected in this study.

### 4.2. Localization

Notably, the trunk localization was the most frequent primary melanoma site in both groups. Melanoma arising in the trunk is linked to intermittent, recreational ultraviolet radiation (UVR) exposure rather than chronic occupational exposure. Site-specific melanoma risk models suggest that head and neck melanomas correlate more strongly with chronic UVR exposure markers such as actinic keratosis, whereas trunk melanomas are associated more with intermittent UVR exposure and higher acquired nevus burden [35,36,37]. Truncal lesions may also be less noticeable by patients compared with lesions on areas such as the face or hands, potentially contributing to delayed presentation, particularly in older individuals or in those with limited access to routine skin examinations and screening programs.

### 4.3. Histopathologic Characteristics of Melanomas

The most clinically relevant finding in our study sample was the distribution of histologic subtypes and tumor thickness. While superficial spreading melanoma predominated in Belgrade, nodular melanoma was the most common subtype in Montenegro, accompanied by significantly higher median Breslow thickness and a greater proportion of very thick melanomas (>4 mm). Superficial spreading melanoma typically progresses through an initial radial growth phase, during which malignant melanocytes spread horizontally within the epidermis with low metastatic potential, before eventually transitioning to vertical invasion. Nodular melanoma, on the other hand, largely bypasses this radial phase and enters the vertical growth phase from onset, resulting in earlier dermal invasion and greater tumor thickness at the time of clinical detection [25,38]. This intrinsic biological difference could also contribute to the higher median Breslow thickness observed in the Montenegrin cohort.

However, the reason for the higher proportion of nodular melanoma in Montenegro is not immediately clear based on ambient sun exposure alone. Based on findings by Micu et al., nodular melanomas exhibit weaker or non-significant latitude gradients, which supports the possibility that factors such as health-seeking behavior, access to dermatologic care, different diagnostic or referral patterns may play an important role in subtype distribution and Breslow thickness at presentation [39]. An alternative explanation relates to the divergent pathway hypothesis of melanoma genesis. According to this hypothesis, melanomas arise through two distinct routes: one associated with a nevus-prone phenotype and intermittent ultraviolet exposure, and another with chronic cumulative UV exposure and a lower nevus count, independent ambient sunshine hours [40]. Differences in population-based nevus phenotype or UV exposure patterns between the two cohorts could therefore also contribute to the observed subtype distribution. Since the relevant data (nevus count and phenotype data) were not available and included in this study, the hypothesis could not be formally tested. Our multivariable analysis further demonstrated that older age and male sex were independently associated with higher Breslow thickness categories. According to the current data, melanomas in older individuals and men are more likely to present with adverse features, potentially due to a combination of biological differences, differences in anatomical distribution, and delays in detection or presentation [28,29]. Together, these findings emphasize the influence of host characteristics, tumor growth dynamics and healthcare access on tumor thickness at presentation.

### 4.4. Melanoma Diagnostic and Treatment Pathways

Surgical management of melanoma in Montenegro is done by a single center whereas in Serbia it is done by a larger network of centers providing both surgical and oncologic care. Centralization of care, such as in Montenegro, can introduce some barriers (travel distance, longer waiting times, scheduling issues) that could contribute to delayed assessment and treatment in some patients. Although not directly measured in this study, prolonged waiting times inherent to a centralized referral system may contribute to delayed definitive treatment, which could contribute to increased tumor thickness at the time of surgery. Since this remains just a hypothesis-generating observation, future prospective studies including referral intervals and waiting time are needed to formally test it. Delays, both in diagnosis and treatment, have been associated with worse melanoma outcomes, as also noted by Xiong et al. [41]. Disruptions in access to care such as during the COVID-19 pandemic, have been linked to later stage upon diagnosis and thicker primary lesions [42,43]. Although this study did not directly measure time from lesion onset to diagnosis and treatment, or referral intervals, the observed results in multivariable model support the importance of system- and pathway-level factors in addition to tumor biology itself. Although the study did not evaluate sentinel lymph node (SLN) outcomes or survival endpoints in the analysis, the thickness differences between cohorts could have clinically meaningful implications because Breslow thickness is tightly linked to SLN positivity and prognosis. As supportive evidence, the author previously reported substantially greater Breslow thickness among SLN-positive patients compared with SLN-negative patients, underscoring the importance of clinical correlation in SLNB candidate selection [44]. Overall, the observed results suggest that the Montenegrin group may have presented with a higher risk disease at the time of diagnosis, impacting long-term outcomes. These results support the implementation of targeted public health initiatives focused on earlier detection, prompt diagnostics, and streamlined referral systems.

### 4.5. Strengths and Limitations of the Study

The study’s strengths include its multicentric design across two national referral centers and the use of consistent AJCC thickness categorization with standardized comparative analyses. Nevertheless, several limitations should be acknowledged. The retrospective nature of the study limits control over missing data and confounding factors. The Serbian cohort does not constitute all newly diagnosed melanomas in Serbia, as patients may be diagnosed and treated across multiple regional centers. Therefore, the sample could be subject to selection bias and may over-represent patients referred to a tertiary center. Additionally, no prior sample size calculation was performed, as this was a retrospective study including all consecutively treated patients over a fixed period rather than a prospectively designed comparison, which may limit the ability to detect smaller effect sizes in secondary analyses. Histopathological assessment at each center was performed by a single pathologist experienced in melanocytic lesions, without independent double-reading or formal interobserver variability testing. This is a common limitation of retrospective multicenter studies and may have introduced center-specific reporting differences. Several variables which would test the access of care hypothesis (patient and referral delay, time to surgical excision, education, socioeconomic factors, screening practices) were not included in this study. The study did not include survival analysis, SLNB findings, the use of adjuvant systemic therapy, or recurrence patterns. These variables would strengthen the causal relationship between prognosis and care pathways. Finally, sunshine hours per region represent ecological exposure measures and do not capture individual UVR dose, intermittent exposure patterns, history of sunburn, indoor tanning or any protective behavior. Therefore, the overall results should be interpreted with caution.

### 4.6. Future Directions

Future prospective studies should include patient- and referral-interval data, sentinel lymph node biopsy findings, survival outcomes, recurrence patterns, and the use of adjuvant systemic therapy. Other histopathological features such as ulceration and lymphovascular invasion, while not assessed in this retrospective study, are similarly recognized prognostic determinants and warrant inclusion in future analyses. Additional factors to consider include socioeconomic status, patient health-seeking behavior, screening participation, histopathological review consistency, and molecular biomarkers. Such data would allow direct testing of the healthcare-access hypothesis proposed in this study and clarify its relationship to long-term prognosis of melanoma patients.

## 5. Conclusions

This multicenter cohort study identified meaningful differences in clinical and histopathological melanoma presentation between two neighboring countries. Montenegro demonstrated a higher proportion of nodular melanoma and significantly thicker tumors at diagnosis, and treatment location remained independently associated with higher Breslow thickness categories after adjustment for age and sex. While differences in ambient sun exposure may contribute to regional variability, the observed differences are likely multifactorial and may reflect variations in healthcare accessibility, referral structures, and diagnostic pathways. Future prospective and registry-based studies including time-to diagnosis, geographic accessibility variables and survival outcomes are needed to clarify the underlying causes of these differences and to guide future targeted health interventions aimed at early detection and improved melanoma care pathways.

## Figures and Tables

**Table 1:** Demographic and clinical characteristics of the overall study population; SSM—superficial spreading melanoma; LMM—lentigo maligna melanoma; ALM—acral lentiginous melanoma; UCCS—University Clinical Center of Serbia; CCM—Clinical Center of Montenegro.

Variables	*n* = 412 (100%)
Age (mean ± SD)	62.88 ± 16.28
Age groups:	
≤40	46 (11.2%)
41–60	119 (28.9%)
61–80	205 (49.8%)
81+	42 (10.2%)
Sex:	
Male	215 (52.2%)
Female	197 (47.8%)
Treating Institution:	
UCCS	229 (55.6%)
CCM	183 (44.4%)
Primary melanoma localization:	
Head and neck	73 (17.7%)
Trunk	156 (37.9%)
Upper extremities	75 (18.2%)
Lower extremities	98 (23.8%)
Acral	10 (2.4%)
Melanoma subtype:	
SSM	222 (53.9%)
Nodular	147 (35.7%)
LMM	16 (3.9%)
ALM	10 (2.4%)
Other	17 (4.1%)
Breslow thickness (median)	2.4 (0.2–90)
Breslow thickness categories:	
≤1 mm	115 (27.9%)
1.01–2.0 mm	72 (17.5%)
2.01–4.0 mm	91 (22.1%)
>4.0 mm	134 (32.5%)

**Table 2:** Comparison of demographic, clinical, and histopathological characteristics between patients treated at UCCS and CCM. Continuous variables are expressed as mean ± SD or median (range), and categorical variables as number (percentage); UCCS—University Clinical Center of Serbia; CCM—Clinical Center of Montenegro.

Variables	UCCS	CCM	*p* Value
	*n* = 229 (55.6%)	*n* = 183 (44.4%)	
Age (mean ± SD)	63.07 ± 15.45	62.66 ± 17.31	0.802
Age groups:			0.228
≤40	27 (11.8%)	19 (10.4%)	
41–60	57 (24.9%)	62 (33.9%)	
61–80	120 (52.4%)	85 (46.4%)	
81+	25 (10.9%)	17 (9.3%)	
Sex:			0.921
Male	119 (52.0%)	96 (52.5%)	
Female	110 (48.0%)	87 (47.5%)	
Sunny hours per year (median):	2112	2238	<0.001
Primary melanoma localization:			0.970
Head and neck	41 (17.9%)	32 (17.5%)	
Trunk	88 (38.5%)	68 (37.1%)	
Upper extremities	39 (17.0%)	36 (19.7%)	
Lower extremities	55 (24.0%)	43 (23.5%)	
Acral	6 (2.6%)	4 (2.2%)	
Melanoma subtype:			<0.001
SSM	150 (65.6%)	72 (39.3%)	
Nodular	47 (20.5%)	100 (54.7%)	
LMM	14 (6.1%)	2 (1.1%)	
ALM	6 (2.6%)	4 (2.2%)	
Other	12 (5.2%)	5 (2.7%)	
Breslow thickness (median):	2 (0.2–55)	3 (0.2–90)	0.001
Breslow thickness categories:			0.001
≤1 mm	82 (35.8%)	33 (18.0%)	
1.01–2.0 mm	35 (15.3%)	37 (20.2%)	
2.01–4.0 mm	47 (20.5%)	44 (24.0%)	
>4.0 mm	65 (28.4%)	69 (37.8%)	

**Table 3:** Multivariable ordinal logistic regression analysis evaluating independent predictors of higher Breslow thickness categories; OR—odds ratios, CI—confidence intervals, UCCS—University Clinical Center of Serbia; CCM—Clinical Center of Montenegro.

Variable	OR	95% CI for OR	*p*
Age	1.03	1.02–1.04	<0.001
Sex (male vs. female)	1.50	1.05–2.16	0.027
Institution (CCM vs. UCCS)	1.87	1.31–2.68	0.001
Localization	1.22	0.85–1.75	0.287

## Data Availability

All data is contained within the article.
